# Crystal structure of poly[[di-μ_3_-acetato-tetra­aqua­bis­(μ_2_-cyclo­hexane-1,4-di­carboxyl­ato)dilanth­an­um(III)] dihydrate]

**DOI:** 10.1107/S2056989017016103

**Published:** 2017-11-21

**Authors:** R. Drisya, U. S. Soumya Mol, P. R. Satheesh Chandran, M. Sithambaresan, M. R. Sudarsankumar

**Affiliations:** a Department of Chemistry, Mahatma Gandhi College, Thiruvananthapuram 695 004, Kerala, India; b Department of Chemistry, Faculty of Science, Eastern University, Sri Lanka, Chenkalady, Sri Lanka

**Keywords:** crystal structure, cyclo­hexane-1,4-di­carb­oxy­lic acid, lanthanum(III) complex

## Abstract

The title compound, {[La_2_(CH_3_COO)_2_(C_8_H_10_O_4_)_2_(H_2_O)_4_]·2H_2_O}_*n*_ or [La_2_(*ac*)_2_(*e,a*-*cis*-1,4-chdc)_2_(H_2_O)_4_]·2H_2_O, where *ac* is acetate and 1,4-chdc is cyclo­hexane-1,4-di­carboxyl­ate anion, is a binuclear lanthanum(III) complex. Each metal atom is deca­coordinated by four O atoms from two distinct 1,4-chdc^2−^ ligands, four O atoms from three acetate groups and two O atoms from coordinated water mol­ecules to form a distorted bicapped square-anti­prismatic geometry. Two non-coordinated water mol­ecules are also present in the formula unit. The most remarkable feature of this compound is that it possesses a only *cis* conformation for cyclo­hexane-1,4-di­carb­oxy­lic acid, although the raw material consists of a mixture of *cis* and *trans* isomers. The μ_3_-η^2^:η^2^ coordination mode of the bridging acetate group and the flexible di­carboxyl­ate fragments of 1,4-chdc^2−^ results in the formation of infinite two-dimensional lanthanide–carboxyl­ate layers within the crystal structure. The directionality of strong inter­molecular O—H⋯O and weak C—H⋯O inter­actions provides robustness to the layers, which leads to the construction of a three-dimensional supra­molecular network. The crystal studied was refined as a two-component twin.

## Abstract 2

The title compound is a binuclear lanthanum(III) complex having each metal ion in deca­coordination with oxygen atoms from 1,4-chdc^2−^ ligands, acetate groups and coordinated water mol­ecules to form a distorted bicapped square anti­prismatic geometry. The strong inter­molecular O–H⋯O and weak C–H⋯O inter­actions lead to the construction of a three-dimensional supra­molecular architecture.

## Chemical context   

1,4-Cyclo­hexa­nedi­carboxyic acid (1,4-chdcH_2_) is a flexible alicyclic, ditopic ligand having a chair-type backbone structure, which has been used for the construction of many coord­ination polymers (CPs) with remarkable architectures (Liu *et al.*, 2010 ▸). It can exist in three different conformations – two *trans* isomers, (*a,a*) and (*e,e*), and one *cis (e,a)* form. From a thermodynamical point of view, the *trans* (*e,e*) form is the most stable of the three different conformations as a result of the equatorial–equatorial –COOH groups and the *trans* (*a,a*) isomer is the least stable because of 1,3-diaxial hindrance (Yu *et al.*, 2007 ▸; Gong *et al.*, 2005 ▸; Bi *et al.*, 2003 ▸; Du *et al.*, 2005 ▸; Chen *et al.*, 2014 ▸)·Theoretical calculations suggest that the isomers tend to cause conformational inversion within the ligand structure due to the flexibility of the C—C bond rotation and also because of the extremely low free energy change between them (Qiblawi *et al.*, 2013 ▸; Lin & Tong, 2011 ▸; Liu *et al.*, 2010 ▸). Furthermore, the isomeric separation of the organic ligand can be controlled by several factors such as the pH of the solution, the nature of the metal ion, the co-ligand, the reaction solvent and the temperature (Lin & Tong, 2011 ▸; Liu *et al.*, 2010 ▸).
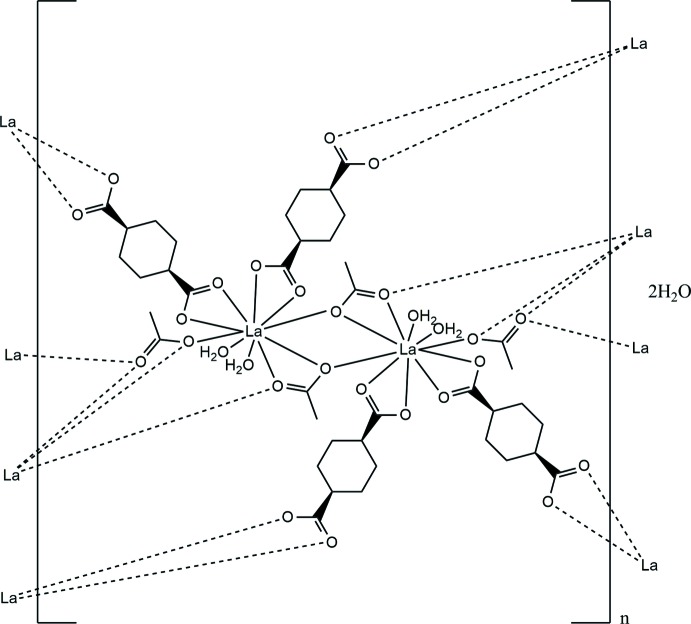


## Structural commentary   

**Figure 1:**
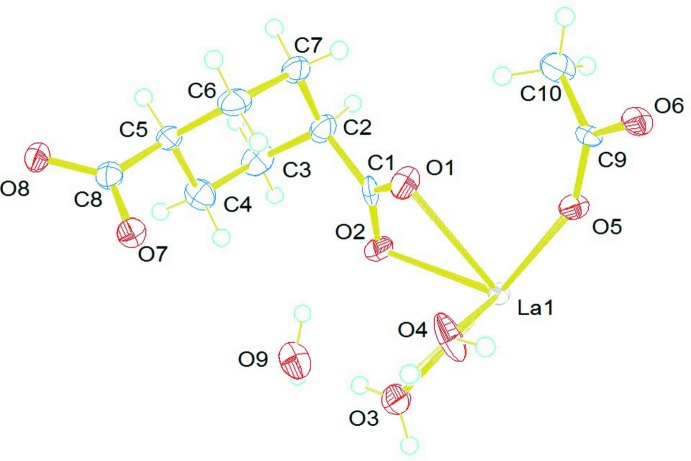
*ORTEP* view of the mol­ecular structure of the title complex with the atom-numbering scheme and ellipsoids drawn at the 50% probability level..

**Figure 2:**
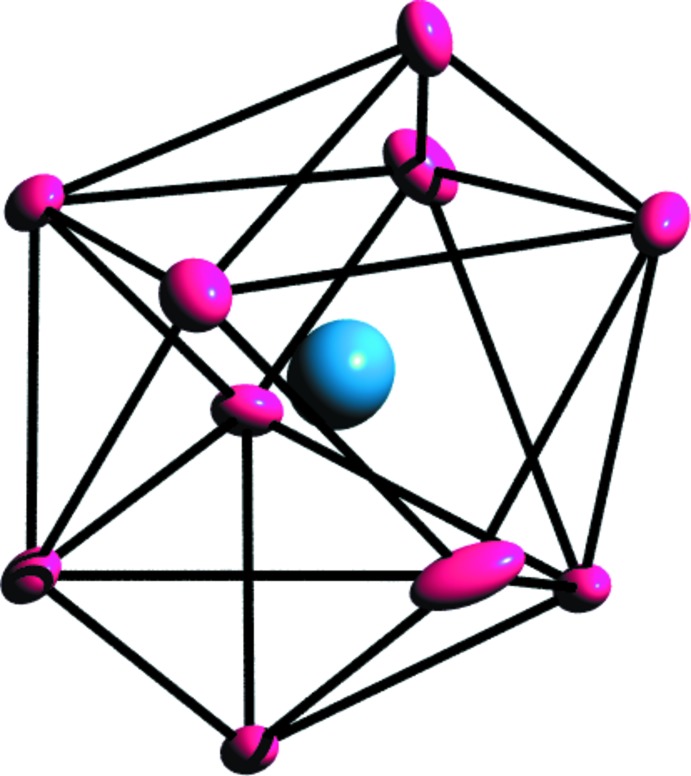
Bicapped square-anti­prismatic geometry of an [LaO_10_] polyhedron. Displacement ellipsoids are drawn at the 80% probability level.

The asymmetric unit of the title compound consists of one crystallographically unique La metal ion, a fully deprotonated 1,4-chdc^2−^ anion, an acetate moiety and three water mol­ecules (two coordinated and one non-coordinated). From the mol­ecular structure (Fig. 1 ▸), it is evident that each La^III^ atom has a distorted bicapped square-anti­prismatic coordination sphere defined by four oxygen atoms from two distinct 1,4-chdc^2−^ ligands (O1, O2, O7, O8), four oxygen atoms from three acetate groups (O5, O6, O5′, O6′) and two oxygen atoms from coordinated water mol­ecules (O3, O4) to form a [LaO_10_] coordination polyhedron (Fig. 2 ▸). Of the three prevalent conformations of 1,4-chdcH_2_, low temperature usually favours the *cis* (*e,a*) and high temperature favours the trans (*e,e*) conformational compounds (Lin & Tong, 2011 ▸; Lu *et al.*, 2008 ▸; Bi *et al.*, 2004 ▸). Here, the bent structure of the organic linker possesses an L-shaped *cis* (*e,a*) conformation within the crystal structure. The corresponding La—O bond lengths are in the range 2.506 (8)—2.792 (7) Å and the O—La—O bond angles vary from 46.51 (19) to 170.7 (2)°. The La—O bond distances are comparable with those in several reported structures in which 1,4-cyclo­hexa­nedi­carb­oxy­lic acid exists in various coordination modes and conformations (Rao *et al.*, 2007 ▸; Qi *et al.*, 2008 ▸).

**Figure 3:**
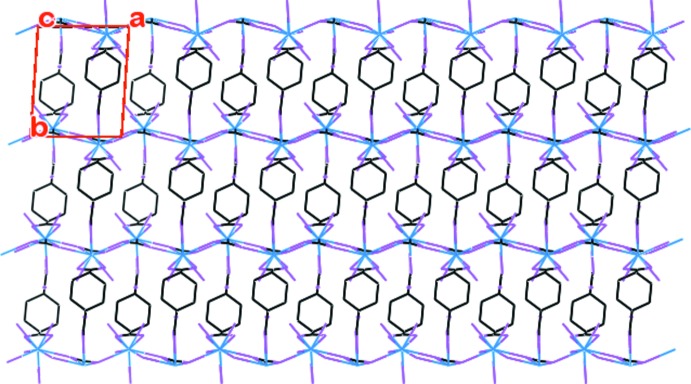
Perspective view of the packing along the *c* axis.

**Figure 4:**
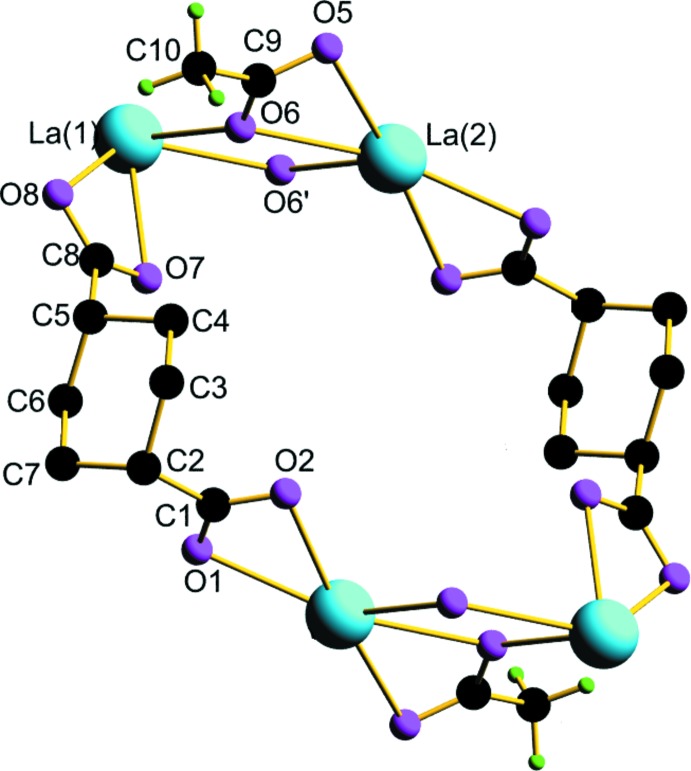
The 24-membered macrocyclic ring formation by 1,4-chdc^2−^ between two [La_2_O_2_] chains.

The bridging μ_3_-η^2^ **:**η^2^ coordination mode (each oxygen atom connects two metal atoms) of the acetate group joins two [LaO_10_] polyhedra by edge sharing to form a dimeric structure. The dimers are then inter­linked by La—O—La bonding and as a consequence of this, infinite zigzag 1D [La_2_O_2_] chains are formed. Within these chains, La**⋯**La non-bonding distances are found to be 4.5835 (9) and 4.4125 (9) Å. Additionally, the *bis*-bidentate chelating μ_2_-η^1^ **:**η^1^ **:**η^1^ **:**η^1^ coordination mode of the di­carboxyl­ate group of 1,4-chdc^2−^ connects two metal atoms and hence converts it into a 2D coordination polymeric structure parallel to the *ab* plane. A perspective view of the packing along the *c* axis in a wireframe model (Fig. 3 ▸) shows the formation of infinite 2D lanthanide–carboxyl­ate layers. The [La_2_O_2_] chains are then further inter­connected by a di­carboxyl­ate anion from two 1,4-chdc^2−^ units to form a 24-membered macrocyclic ring as shown in Fig. 4 ▸. A series of organotin complexes of the *cis* and *trans* isomers of 1,4-chdcH_2_ show similar 2D networks containing 26- and 36-membered tetra­tin macrocyclic rings (Ma *et al.*, 2009 ▸).

## Supra­molecular features   

**Figure 5:**
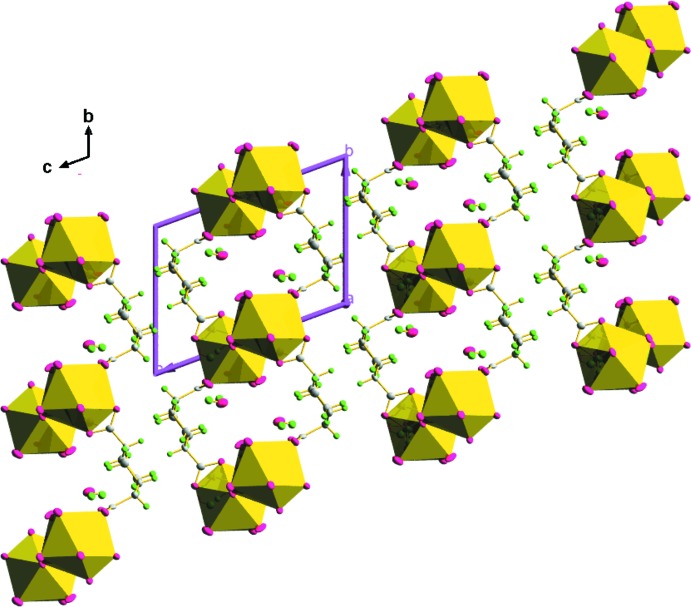
Polyhedral view along the *a* axis showing the free water mol­ecules.

**Figure 6:**
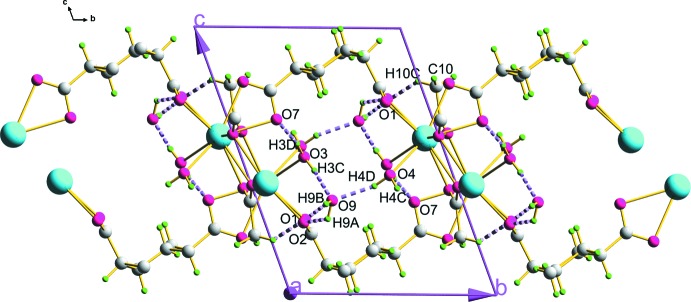
Hydrogen-bonding inter­actions (dashed lines) in the structure of the title compound. For symmetry operations, see Table 1 ▸.

From the polyhedral view along the *a* axis (Fig. 5 ▸), it is clear that the two lattice water mol­ecules residing in the voids of the 1,4-chdc^2−^ units are responsible for the development of hydro­philic channels within the crystal structure. The hydrogen-bonding inter­actions (Table 1 ▸) shown in Fig. 6 ▸ play a vital role in increasing the stability and higher dimensionality of the crystal packing. Here, the oxygen atom O9 of the lattice water mol­ecule acts as a donor for hydrogen bonds with oxygen atoms O1 and O2 of the carboxyl­ate group of the 1,4-chdc^2−^ ligand [O9—H9*A*⋯O2 = 2.786 (12) Å and O9—H9*B*⋯O1^iii^ = 2.846 (11) Å]. It also acts as the hydrogen-bond acceptor for oxygen atoms O3 and O4 of the coordinated water mol­ecules [O3—H3*C*⋯O9 = 2.858 (12) Å and O4—H4*D*⋯O9^ii^ 2.812 (11) Å]. Similarly, oxygen atom O7 of the carboxyl­ate group of 1,4-chdc acts as an acceptor to atoms O3 and O4 of the coordinated water mol­ecules [O3—H3*D*⋯O7^ii^ = 2.750 (11) Å and O4—H4*C*⋯O7^i^ = 2.771 (10) Å]. Apart from this strong inter­molecular hydrogen bonding, there are also weak C—H⋯O inter­actions between the carbon atom C10 of the coordinated acetate group and the O1 oxygen atom of a carboxyl­ate group of the organic linker [C10—H10*C*⋯O1 = 3.295 (14) Å].

## Database survey   

In the three-dimensional structures of [La_2_(1,4-chdc)_3_(H_2_O)_4_], [La_3_(1,4-Hchdc)_2_(1,4-chdc)_5_(H_2_O)_2_]·H_2_O and [La_2_(1,4-chdc)_3_(H_2_O)]·2.5H_2_O, the di­carboxyl­ate anion exists in different conformations obtained under hydro­thermal conditions (Rao *et al.*, 2007 ▸). Similarly a two-dimensional lanthanum coordin­ation polymer [La_2_(1,10-phen)_2_(1,4-chdc)_3_]·2.5H_2_O with π–π stacking was observed by the incorporation of 1,10-phenanthroline as a co-ligand along with 1,4-cyclo­hexa­nedi­carb­oxy­lic acid (Qi *et al.*, 2008 ▸). Additionally, dimethyl formamide (DMF) and dimethyl sulfoxide (DMSO) solvent-coordinated lanthanum complexes, one-dimensional [La(*cis*-chdc)(DMF)_2_(NO_3_)] and three-dimensional [La_2_(*trans*-chdc)_3_(DMSO)_4_] have also been reported. The presence of solvent mol­ecules can completely segregate the *cis* and *trans* conformations of 1,4-chdc (Tian *et al.*, 2009 ▸).

## Synthesis and crystallization   

Single crystals of the title compound were prepared by the gel-diffusion technique at ambient temperature using sodium metasilicate nona­hydrate (Na_2_S_2_O_3_·9H_2_O) as the gel medium. The optimum condition for crystal growth was obtained by dissolving 0.75 g of 1,4-H_2_chdc in 25 ml of 1.04 g cm^−3^ dense gel medium. 5 ml of the above solution was poured into glass tubes and the pH of the solution was set to 7.0 by adding glacial acetic acid drop by drop. On completion of the gel-setting process, 3 ml of 0.5 *M* concentration of aqueous lanthanum nitrate solution was added as the upper reagent. The whole arrangement was kept undisturbed at room temperature and was covered to protect it from the foreign matter present in the atmosphere. Within seven days, transparent, colourless block-shaped crystals were observed at the gel inter­face. The diffusion of La^3+^ ions and 1,4-chdcH_2_ through the fine pores of the gel media lead to the expected chemical reaction as shown below:

**2La(NO_3_)_3_·6H_2_O + 2C_8_H_12_O_4_ + 2CH_3_COOH** → **[La_2_(CH_3_COO)_2_(C_8_H_10_O_4_)_2_(H_2_O)_4_]·2H_2_O + 6HNO_3_.**

Elemental analysis calculated (%) for C_20_H_38_La_2_O_18_ (844.32): C, 28.42; H, 4.50. Found (%): C, 28.36; H, 4.33. IR (KBr, cm^−1^): 3380, 2940, 1573, 1460, 743, 673, 597.

## Refinement   

Crystal data, data collection and structure refinement details are summarized in Table 2 ▸. Carbon-bound hydrogen atoms were placed in calculated positions and included in the refinement in the riding-model approximation with C—H distances of 0.96–0.98 Å and with *U*_iso_(H) = 1.2*U*_eq_(C) for methyl hydrogen atoms and *U*_iso_(H) = 1.2*U*_eq_(C) for all others. Water hydrogen atoms were located from difference-Fourier maps and refined with an O—H distance restraint of 0.90 (2) Å and an H⋯H separation of 1.39 (2) Å. The isotropic displacement parameters of the hydrogen atoms attached to atoms O3, O4 and O9 were made equal by using an EDAP instruction. The crystal studied was refined as a two-component twin (BASF = 0.4203).

## Supplementary Material

Crystal structure: contains datablock(s) I. DOI: 10.1107/S2056989017016103/vn2132sup1.cif

Structure factors: contains datablock(s) I. DOI: 10.1107/S2056989017016103/vn2132Isup2.hkl

CCDC reference: 1546730

Additional supporting information:  crystallographic information; 3D view; checkCIF report

## Figures and Tables

**Table 1:** Hydrogen-bond geometry (Å, °)

*D*—H⋯*A*	*D*—H	H⋯*A*	*D*⋯*A*	*D*—H⋯*A*
C10—H10*C*⋯O1	0.96	2.49	3.295 (14)	141
O4—H4*C*⋯O7^i^	0.90 (2)	1.92 (5)	2.771 (10)	158 (12)
O4—H4*D*⋯O9^ii^	0.89 (2)	1.96 (6)	2.812 (11)	158 (12)
O3—H3*C*⋯O9	0.90 (2)	1.97 (3)	2.858 (12)	172 (13)
O3—H3*D*⋯O7^ii^	0.90 (2)	1.86 (3)	2.750 (11)	170 (14)
O9—H9*A*⋯O2	0.90 (2)	2.20 (11)	2.786 (12)	122 (11)
O9—H9*B*⋯O1^iii^	0.90 (2)	1.97 (5)	2.846 (11)	164 (13)

Symmetry codes: (i) 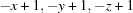; (ii) 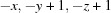; (iii) 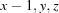.

**Table 2:** Experimental details

Crystal data	
Chemical formula	[La_2_(C_2_H_3_O_2_)_2_(C_8_H_10_O_4_)_2_(H_2_O)_4_]·2H_2_O
*M*_r_	844.32
Crystal system, space group	Triclinic, *P* 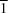
Temperature (K)	293
*a*, *b*, *c* (Å)	6.9341 (8), 8.9597 (13), 12.3030 (16)
α, β, γ (°)	110.217 (5), 91.060 (5), 93.280 (5)
*V* (Å^3^)	715.49 (16)
*Z*	1
Radiation type	Mo *K*α
μ (mm^−1^)	3.02
Crystal size (mm)	0.20 × 0.15 × 0.15

Data collection	
Diffractometer	Bruker Kappa APEXII CCD
Absorption correction	Multi-scan (*SADABS*; Bruker, 2004 ▸)
*T*_min_, *T*_max_	0.60, 0.74
No. of measured, independent and observed [*I* > 2σ(*I*)] reflections	2818, 2815, 2447
(sin θ/λ)_max_ (Å^−1^)	0.617

Refinement	
*R*[*F* ^2^ > 2σ(*F* ^2^)], *wR*(*F* ^2^), *S*	0.041, 0.146, 1.07
No. of reflections	2818
No. of parameters	204
No. of restraints	9
H-atom treatment	H atoms treated by a mixture of independent and constrained refinement
Δρ_max_, Δρ_min_ (e Å^−3^)	2.17, −2.46

Computer programs: *APEX2*, *SAINT* and *XPREP* (Bruker, 2004 ▸), *SIR92* (Sheldrick, 2015 ▸), *DIAMOND* (Brandenburg, 2010 ▸), *SHELXL2014* (Sheldrick, 2015 ▸) and *publCIF* (Westrip, 2010 ▸).

## supplementary crystallographic information

### Crystal data

**Table:**

[La_2_(C_2_H_3_O_2_)_2_(C_8_H_10_O_4_)_2_(H_2_O)_4_]·2H_2_O	*Z* = 1
*M**_r_* = 844.32	*F*(000) = 416
Triclinic, *P*1	*D*_x_ = 1.960 Mg m^−^^3^
*a* = 6.9341 (8) Å	Mo *K*α radiation, λ = 0.71073 Å
*b* = 8.9597 (13) Å	Cell parameters from 7100 reflections
*c* = 12.3030 (16) Å	θ = 2.8–30.9°
α = 110.217 (5)°	µ = 3.02 mm^−^^1^
β = 91.060 (5)°	*T* = 293 K
γ = 93.280 (5)°	Block, colourless
*V* = 715.49 (16) Å^3^	0.20 × 0.15 × 0.15 mm

### Data collection

**Table:**

Bruker Kappa APEXII CCD diffractometer	2815 independent reflections
Radiation source: Sealed tube	2447 reflections with *I* > 2σ(*I*)
ω and φ scan	θ_max_ = 26.0°, θ_min_ = 2.4°
Absorption correction: multi-scan (SADABS; Bruker, 2004)	*h* = −8→8
*T*_min_ = 0.60, *T*_max_ = 0.74	*k* = −11→10
2818 measured reflections	*l* = 0→15

### Refinement

**Table:**

Refinement on *F*^2^	9 restraints
Least-squares matrix: full	Hydrogen site location: mixed
*R*[*F*^2^ > 2σ(*F*^2^)] = 0.041	H atoms treated by a mixture of independent and constrained refinement
*wR*(*F*^2^) = 0.146	*w* = 1/[σ^2^(*F*_o_^2^) + (0.0821*P*)^2^ + 6.1459*P*] where *P* = (*F*_o_^2^ + 2*F*_c_^2^)/3
*S* = 1.07	(Δ/σ)_max_ = 0.005
2818 reflections	Δρ_max_ = 2.17 e Å^−^^3^
204 parameters	Δρ_min_ = −2.46 e Å^−^^3^

### Special details

**Table:**

Geometry. All esds (except the esd in the dihedral angle between two l.s. planes) are estimated using the full covariance matrix. The cell esds are taken into account individually in the estimation of esds in distances, angles and torsion angles; correlations between esds in cell parameters are only used when they are defined by crystal symmetry. An approximate (isotropic) treatment of cell esds is used for estimating esds involving l.s. planes.
Refinement. Refined as a two-component twin.

### Fractional atomic coordinates and isotropic or equivalent isotropic displacement parameters (Å^2^)

**Table:**

	*x*	*y*	*z*	*U*_iso_*/*U*_eq_	
C1	0.2377 (17)	0.7936 (10)	0.7752 (7)	0.0203 (18)	
C2	0.2486 (18)	0.7706 (11)	0.8920 (8)	0.030 (2)	
H2	0.2447	0.8760	0.9522	0.036*	
C3	0.0724 (17)	0.6681 (16)	0.9058 (11)	0.033 (3)	
H3A	0.0698	0.6715	0.9855	0.040*	
H3B	−0.0448	0.7116	0.8889	0.040*	
C4	0.0778 (17)	0.4962 (14)	0.8253 (10)	0.030 (3)	
H4A	0.0694	0.4919	0.7455	0.036*	
H4B	−0.0331	0.4339	0.8379	0.036*	
C5	0.2631 (16)	0.4239 (11)	0.8460 (7)	0.024 (2)	
H5	0.2633	0.4196	0.9245	0.028*	
C6	0.4375 (16)	0.5264 (15)	0.8372 (11)	0.030 (3)	
H6A	0.4468	0.5213	0.7574	0.036*	
H6B	0.5526	0.4836	0.8576	0.036*	
C7	0.4325 (17)	0.6994 (14)	0.9147 (10)	0.028 (2)	
H7A	0.5438	0.7602	0.9010	0.034*	
H7B	0.4403	0.7067	0.9952	0.034*	
C8	0.2736 (16)	0.2574 (10)	0.7614 (8)	0.0229 (19)	
C9	0.7522 (14)	1.0347 (9)	0.6555 (7)	0.0141 (16)	
C10	0.7542 (18)	1.0729 (13)	0.7831 (8)	0.030 (2)	
H10A	0.7030	1.1749	0.8194	0.046*	
H10B	0.8846	1.0758	0.8118	0.046*	
H10C	0.6763	0.9926	0.8006	0.046*	
O1	0.3916 (11)	0.7991 (10)	0.7225 (7)	0.0280 (18)	
O2	0.0804 (11)	0.8116 (10)	0.7334 (7)	0.0272 (18)	
O3	−0.0144 (11)	0.6946 (10)	0.4871 (7)	0.0310 (18)	
O4	0.3865 (13)	0.6914 (10)	0.4470 (8)	0.039 (2)	
O5	0.5999 (10)	0.9978 (10)	0.5957 (6)	0.0219 (16)	
O6	0.9100 (10)	1.0455 (9)	0.6066 (6)	0.0220 (16)	
O7	0.2827 (11)	0.2366 (7)	0.6530 (5)	0.0223 (14)	
O8	0.2635 (12)	0.1397 (7)	0.7932 (5)	0.0283 (15)	
O9	−0.2682 (13)	0.6345 (9)	0.6499 (7)	0.0384 (18)	
La1	0.23952 (8)	0.92768 (5)	0.58722 (4)	0.01486 (18)	
H4C	0.474 (15)	0.704 (14)	0.398 (8)	0.05 (3)*	
H4D	0.326 (16)	0.597 (8)	0.406 (8)	0.05 (3)*	
H3C	−0.088 (14)	0.668 (17)	0.538 (9)	0.06 (3)*	
H3D	−0.105 (12)	0.705 (17)	0.437 (9)	0.06 (3)*	
H9A	−0.207 (15)	0.687 (15)	0.719 (6)	0.06 (3)*	
H9B	−0.388 (9)	0.669 (17)	0.663 (10)	0.06 (3)*	

### Atomic displacement parameters (Å^2^)

**Table:**

	*U*^11^	*U*^22^	*U*^33^	*U*^12^	*U*^13^	*U*^23^
C1	0.026 (5)	0.012 (4)	0.025 (4)	−0.004 (4)	0.006 (5)	0.008 (3)
C2	0.047 (7)	0.023 (5)	0.019 (4)	0.003 (5)	−0.002 (5)	0.008 (4)
C3	0.029 (7)	0.041 (7)	0.034 (7)	0.011 (5)	0.007 (5)	0.017 (6)
C4	0.031 (7)	0.027 (6)	0.030 (6)	0.004 (5)	0.003 (4)	0.005 (5)
C5	0.030 (6)	0.026 (5)	0.016 (4)	−0.005 (4)	−0.002 (4)	0.011 (4)
C6	0.022 (6)	0.037 (7)	0.033 (6)	0.004 (5)	−0.005 (4)	0.014 (5)
C7	0.041 (8)	0.025 (6)	0.017 (5)	0.001 (5)	−0.004 (4)	0.007 (4)
C8	0.017 (5)	0.024 (5)	0.029 (5)	0.002 (4)	−0.001 (4)	0.012 (4)
C9	0.010 (4)	0.012 (3)	0.018 (4)	0.003 (4)	−0.002 (4)	0.001 (3)
C10	0.026 (6)	0.042 (6)	0.020 (4)	−0.006 (5)	0.003 (5)	0.007 (4)
O1	0.016 (4)	0.039 (5)	0.039 (5)	0.005 (3)	0.005 (3)	0.025 (4)
O2	0.026 (5)	0.031 (5)	0.028 (4)	0.004 (3)	−0.005 (3)	0.015 (4)
O3	0.026 (5)	0.035 (5)	0.034 (5)	−0.005 (3)	−0.002 (3)	0.017 (4)
O4	0.033 (5)	0.022 (4)	0.051 (5)	−0.009 (3)	0.023 (4)	−0.001 (4)
O5	0.012 (4)	0.037 (4)	0.020 (4)	−0.001 (3)	0.002 (3)	0.014 (3)
O6	0.011 (4)	0.032 (4)	0.025 (4)	−0.001 (3)	0.002 (3)	0.011 (3)
O7	0.025 (4)	0.024 (3)	0.018 (3)	0.001 (3)	0.006 (3)	0.007 (2)
O8	0.042 (4)	0.021 (3)	0.024 (3)	−0.001 (4)	−0.004 (3)	0.011 (3)
O9	0.023 (5)	0.033 (4)	0.057 (5)	0.004 (4)	0.004 (4)	0.013 (4)
La1	0.0121 (3)	0.0169 (3)	0.0169 (3)	0.0017 (2)	0.0015 (2)	0.00745 (18)

### Geometric parameters (Å, º)

**Table:**

C1—O2	1.240 (13)	C9—La1^ii^	3.119 (8)
C1—O1	1.266 (13)	C10—H10A	0.9600
C1—C2	1.522 (12)	C10—H10B	0.9600
C1—La1	2.952 (8)	C10—H10C	0.9600
C2—C7	1.521 (17)	O1—La1	2.570 (7)
C2—C3	1.533 (17)	O2—La1	2.601 (8)
C2—H2	0.9800	O3—La1	2.588 (8)
C3—C4	1.519 (18)	O3—H3C	0.90 (2)
C3—H3A	0.9700	O3—H3D	0.90 (2)
C3—H3B	0.9700	O4—La1	2.506 (8)
C4—C5	1.527 (16)	O4—H4C	0.90 (2)
C4—H4A	0.9700	O4—H4D	0.89 (2)
C4—H4B	0.9700	O5—La1	2.533 (7)
C5—C8	1.502 (12)	O5—La1^ii^	2.792 (7)
C5—C6	1.505 (15)	O6—La1^iii^	2.552 (7)
C5—H5	0.9800	O6—La1^ii^	2.674 (7)
C6—C7	1.516 (17)	O7—La1^i^	2.598 (6)
C6—H6A	0.9700	O8—La1^i^	2.585 (6)
C6—H6B	0.9700	O9—H9A	0.90 (2)
C7—H7A	0.9700	O9—H9B	0.90 (2)
C7—H7B	0.9700	La1—O6^iv^	2.552 (7)
C8—O8	1.244 (11)	La1—O8^v^	2.585 (6)
C8—O7	1.284 (11)	La1—O7^v^	2.598 (6)
C8—La1^i^	2.983 (9)	La1—O6^ii^	2.674 (7)
C9—O5	1.237 (11)	La1—O5^ii^	2.792 (7)
C9—O6	1.273 (11)	La1—C8^v^	2.983 (9)
C9—C10	1.487 (11)		
			
O2—C1—O1	120.1 (8)	C9—O6—La1^ii^	98.1 (5)
O2—C1—C2	120.4 (9)	La1^iii^—O6—La1^ii^	115.2 (3)
O1—C1—C2	119.5 (10)	C8—O7—La1^i^	94.3 (5)
O2—C1—La1	61.6 (5)	C8—O8—La1^i^	96.0 (5)
O1—C1—La1	60.2 (5)	H9A—O9—H9B	101 (3)
C2—C1—La1	164.8 (6)	O4—La1—O5	73.2 (3)
C1—C2—C7	114.2 (9)	O4—La1—O6^iv^	135.8 (3)
C1—C2—C3	110.9 (9)	O5—La1—O6^iv^	143.4 (2)
C7—C2—C3	109.4 (8)	O4—La1—O1	77.7 (3)
C1—C2—H2	107.4	O5—La1—O1	73.8 (2)
C7—C2—H2	107.4	O6^iv^—La1—O1	126.4 (2)
C3—C2—H2	107.4	O4—La1—O8^v^	146.0 (3)
C4—C3—C2	111.4 (9)	O5—La1—O8^v^	82.5 (3)
C4—C3—H3A	109.4	O6^iv^—La1—O8^v^	76.9 (2)
C2—C3—H3A	109.4	O1—La1—O8^v^	72.8 (2)
C4—C3—H3B	109.4	O4—La1—O3	67.5 (3)
C2—C3—H3B	109.4	O5—La1—O3	140.7 (3)
H3A—C3—H3B	108.0	O6^iv^—La1—O3	73.0 (3)
C3—C4—C5	111.4 (10)	O1—La1—O3	96.1 (3)
C3—C4—H4A	109.4	O8^v^—La1—O3	131.7 (3)
C5—C4—H4A	109.4	O4—La1—O7^v^	138.0 (2)
C3—C4—H4B	109.4	O5—La1—O7^v^	73.7 (2)
C5—C4—H4B	109.4	O6^iv^—La1—O7^v^	69.9 (2)
H4A—C4—H4B	108.0	O1—La1—O7^v^	116.4 (2)
C8—C5—C6	110.0 (9)	O8^v^—La1—O7^v^	49.88 (18)
C8—C5—C4	111.2 (9)	O3—La1—O7^v^	140.6 (2)
C6—C5—C4	110.4 (8)	O4—La1—O2	103.1 (3)
C8—C5—H5	108.4	O5—La1—O2	121.8 (2)
C6—C5—H5	108.4	O6^iv^—La1—O2	78.8 (2)
C4—C5—H5	108.4	O1—La1—O2	49.7 (2)
C5—C6—C7	113.4 (9)	O8^v^—La1—O2	69.9 (2)
C5—C6—H6A	108.9	O3—La1—O2	67.7 (3)
C7—C6—H6A	108.9	O7^v^—La1—O2	116.2 (2)
C5—C6—H6B	108.9	O4—La1—O6^ii^	83.0 (3)
C7—C6—H6B	108.9	O5—La1—O6^ii^	107.7 (2)
H6A—C6—H6B	107.7	O6^iv^—La1—O6^ii^	64.8 (3)
C6—C7—C2	111.4 (9)	O1—La1—O6^ii^	159.3 (3)
C6—C7—H7A	109.3	O8^v^—La1—O6^ii^	127.8 (2)
C2—C7—H7A	109.3	O3—La1—O6^ii^	69.3 (2)
C6—C7—H7B	109.3	O7^v^—La1—O6^ii^	83.2 (2)
C2—C7—H7B	109.3	O2—La1—O6^ii^	129.9 (2)
H7A—C7—H7B	108.0	O4—La1—O5^ii^	68.8 (3)
O8—C8—O7	119.6 (8)	O5—La1—O5^ii^	61.3 (3)
O8—C8—C5	121.6 (8)	O6^iv^—La1—O5^ii^	103.7 (2)
O7—C8—C5	118.7 (7)	O1—La1—O5^ii^	129.6 (2)
O8—C8—La1^i^	59.5 (5)	O8^v^—La1—O5^ii^	119.3 (2)
O7—C8—La1^i^	60.3 (4)	O3—La1—O5^ii^	104.2 (2)
C5—C8—La1^i^	172.5 (8)	O7^v^—La1—O5^ii^	72.9 (2)
O5—C9—O6	118.9 (7)	O2—La1—O5^ii^	170.7 (2)
O5—C9—C10	121.7 (9)	O6^ii^—La1—O5^ii^	46.51 (19)
O6—C9—C10	119.4 (9)	O4—La1—C1	93.6 (3)
O5—C9—La1^ii^	63.3 (4)	O5—La1—C1	97.2 (3)
O6—C9—La1^ii^	58.1 (4)	O6^iv^—La1—C1	101.4 (3)
C10—C9—La1^ii^	161.8 (6)	O1—La1—C1	25.3 (3)
C9—C10—H10A	109.5	O8^v^—La1—C1	65.9 (2)
C9—C10—H10B	109.5	O3—La1—C1	84.1 (3)
H10A—C10—H10B	109.5	O7^v^—La1—C1	115.7 (2)
C9—C10—H10C	109.5	O2—La1—C1	24.8 (3)
H10A—C10—H10C	109.5	O6^ii^—La1—C1	152.5 (3)
H10B—C10—H10C	109.5	O5^ii^—La1—C1	154.8 (3)
C1—O1—La1	94.4 (6)	O4—La1—C8^v^	151.3 (3)
C1—O2—La1	93.6 (6)	O5—La1—C8^v^	78.0 (3)
La1—O3—H3C	113 (9)	O6^iv^—La1—C8^v^	70.6 (3)
La1—O3—H3D	120 (9)	O1—La1—C8^v^	94.8 (3)
H3C—O3—H3D	101 (3)	O8^v^—La1—C8^v^	24.5 (2)
La1—O4—H4C	121 (8)	O3—La1—C8^v^	141.2 (3)
La1—O4—H4D	127 (8)	O7^v^—La1—C8^v^	25.4 (2)
H4C—O4—H4D	102 (3)	O2—La1—C8^v^	92.3 (3)
C9—O5—La1	147.1 (6)	O6^ii^—La1—C8^v^	105.7 (2)
C9—O5—La1^ii^	93.4 (5)	O5^ii^—La1—C8^v^	97.0 (2)
La1—O5—La1^ii^	118.7 (3)	C1—La1—C8^v^	90.4 (2)
C9—O6—La1^iii^	138.0 (6)		
			
O2—C1—C2—C7	161.8 (9)	C4—C5—C8—O7	63.7 (13)
O1—C1—C2—C7	−20.4 (13)	O2—C1—O1—La1	15.4 (9)
La1—C1—C2—C7	−105 (3)	C2—C1—O1—La1	−162.5 (7)
O2—C1—C2—C3	37.7 (12)	O1—C1—O2—La1	−15.2 (9)
O1—C1—C2—C3	−144.5 (9)	C2—C1—O2—La1	162.6 (7)
La1—C1—C2—C3	131 (3)	O6—C9—O5—La1	174.3 (8)
C1—C2—C3—C4	69.6 (12)	C10—C9—O5—La1	−8.2 (16)
C7—C2—C3—C4	−57.2 (11)	La1^ii^—C9—O5—La1	−168.1 (12)
C2—C3—C4—C5	57.2 (12)	O6—C9—O5—La1^ii^	−17.7 (8)
C3—C4—C5—C8	−176.5 (9)	C10—C9—O5—La1^ii^	159.9 (7)
C3—C4—C5—C6	−54.1 (12)	O5—C9—O6—La1^iii^	−124.4 (8)
C8—C5—C6—C7	176.5 (9)	C10—C9—O6—La1^iii^	58.0 (12)
C4—C5—C6—C7	53.4 (11)	La1^ii^—C9—O6—La1^iii^	−143.1 (9)
C5—C6—C7—C2	−55.0 (12)	O5—C9—O6—La1^ii^	18.6 (9)
C1—C2—C7—C6	−69.6 (12)	C10—C9—O6—La1^ii^	−158.9 (7)
C3—C2—C7—C6	55.3 (11)	O8—C8—O7—La1^i^	4.7 (11)
C6—C5—C8—O8	124.8 (11)	C5—C8—O7—La1^i^	−171.5 (9)
C4—C5—C8—O8	−112.5 (12)	O7—C8—O8—La1^i^	−4.8 (11)
C6—C5—C8—O7	−59.0 (13)	C5—C8—O8—La1^i^	171.4 (9)

Symmetry codes: (i) *x*, *y*−1, *z*; (ii) −*x*+1, −*y*+2, −*z*+1; (iii) *x*+1, *y*, *z*; (iv) *x*−1, *y*, *z*; (v) *x*, *y*+1, *z*.

### Hydrogen-bond geometry (Å, º)

**Table:**

*D*—H···*A*	*D*—H	H···*A*	*D*···*A*	*D*—H···*A*
C10—H10*C*···O1	0.96	2.49	3.295 (14)	141
O4—H4*C*···O7^vi^	0.90 (2)	1.92 (5)	2.771 (10)	158 (12)
O4—H4*D*···O9^vii^	0.89 (2)	1.96 (6)	2.812 (11)	158 (12)
O3—H3*C*···O9	0.90 (2)	1.97 (3)	2.858 (12)	172 (13)
O3—H3*D*···O7^vii^	0.90 (2)	1.86 (3)	2.750 (11)	170 (14)
O9—H9*A*···O2	0.90 (2)	2.20 (11)	2.786 (12)	122 (11)
O9—H9*B*···O1^iv^	0.90 (2)	1.97 (5)	2.846 (11)	164 (13)

Symmetry codes: (iv) *x*−1, *y*, *z*; (vi) −*x*+1, −*y*+1, −*z*+1; (vii) −*x*, −*y*+1, −*z*+1.
